# Stable depletion of RUNX1-ETO in Kasumi-1 cells induces expression and enhanced proteolytic activity of Cathepsin G and Neutrophil Elastase

**DOI:** 10.1371/journal.pone.0225977

**Published:** 2019-12-11

**Authors:** Caroline Schoenherr, Katharina Wohlan, Iris Dallmann, Andreas Pich, Jan Hegermann, Arnold Ganser, Denise Hilfiker-Kleiner, Olaf Heidenreich, Michaela Scherr, Matthias Eder

**Affiliations:** 1 Department of Hematology, Hemostasis, Oncology and Stem Cell Transplantation, Hannover Medical School, Hannover, Germany; 2 Department of Toxicology, Research Core Unit Proteomics, Hannover Medical School, Hannover, Germany; 3 Department of Functional and Applied Anatomy, Research Core Unit Electron Microscopy, Hannover Medical School, Hannover, Germany; 4 Department of Cardiology and Angiology, Hannover Medical School, Hannover, Germany; 5 Wolfson Childhood Cancer Research Centre, Northern Institute for Cancer Research, Newcastle University, Newcastle, United Kingdom; 6 Princess Maxima Center for Pediatric Oncology, Utrecht, Netherlands; Wayne State University, UNITED STATES

## Abstract

The oncogenic fusion protein RUNX1-ETO is a product of the t(8;21) translocation and consists of the hematopoietic transcriptional master regulator RUNX1 and the repressor ETO. RUNX1-ETO is found in 10–15% of acute myeloid leukemia and interferes with the expression of genes that are essential for myeloid differentiation. The neutrophil serine protease Cathepsin G is one of the genes suppressed by RUNX1-ETO, but little is known about its impact on the regulation of other lysosomal proteases. By lentiviral transduction of the t(8;21) positive cell line Kasumi-1 with an RUNX1-ETO specific shRNA, we analyzed long-term effects of stable RUNX1-ETO silencing on cellular phenotypes and target gene expression. Stable anti RUNX1-ETO RNAi reduces both proliferation and apoptosis in Kasumi-1 cells. In addition, long-term knockdown of RUNX1-ETO leads to an upregulation of proteolytic activity in Kasumi-1 cells, which may be released *in vitro* upon cell lysis leading to massive degradation of cellular proteins. We therefore propose that protein expression data of RUNX1-ETO-silenced Kasumi-1 cells must be analyzed with caution, as cell lysis conditions can heavily influence the results of studies on protein expression. Next, a mass spectrometry-based approach was used to identify protease cleavage patterns in RUNX1-ETO-depleted Kasumi-1 cells and Neutrophil Elastase has been identified as a RUNX1-ETO candidate target. Finally, proteolytic activity of Neutrophil Elastase and Cathepsin G was functionally confirmed by si/shRNA-mediated knockdown in Kasumi-1 cells.

## Introduction

The translocation t(8;21) is found in 10–15% of acute myeloid leukemia (AML), representing one of the most prevalent chromosomal aberrations associated with AML. Clinically, AML with the translocation t(8;21) is associated with a relatively favorable prognosis at initial diagnosis but not at relapse [1,2]. The resulting oncogenic fusion protein RUNX1-ETO contains the N-terminal RUNT domain of RUNX1 (AML1) and the almost entire ETO (MTG8) protein [3–5]. The oncogenic potential of RUNX1-ETO is based on its ability to deregulate normal RUNX1-dependent gene expression, for which several mechanisms have been described. RUNX1-ETO acts as dominant-negative inhibitor of RUNX1-dependent gene expression by recruiting the corepressor proteins NCoR and SMRT bound to the ETO moiety of the fusion protein [6–8]. NCoR and SMRT can interact with mSin3a and histone deacetylases (HDAC) [9,10], assembling a repressor complex which leads to transcriptional silencing of RUNX1 target genes like *MDR1* [7], *FOS* [11], *CDKN2A* [12] and *BCL2* [13]. However, RUNX1-ETO can also activate gene expression. It recruits the histone acetyl transferase (HAT) p300/CBP complex, facilitating histone acetylation and, more importantly, the acetylation of RUNX1-ETO itself. This results in increased accessibility of regulatory elements and the recruitment of other activating transcription factors, and allows the transactivation of target genes, e.g. *ID1*, *CDKN1A* (p21) and *EGR1* [14]. In addition, a mechanism by which RUNX1-ETO competes with RUNX1 for the binding to a negative regulatory element driving expression of the cell cycle regulator *CCND2* has been recently proposed by Martinez-Soria et al. [15]. Furthermore, RUNX1-ETO can interact with hematopoietic transcription factors like PU.1, C/EBPα, GATA-1 and E2A thereby interfering with their regulatory functions [16–19]. Other binding partners of RUNX1-ETO include proteins of the HDAC, DNA methyltransferase (DNMT) and protein arginine methyltransferase (PRMT) families, which are involved in the modeling of chromatin structure [20–22], and genome-wide changes in transcription factor binding have been shown for the depletion of RUNX1-ETO in AML cells [23]. Despite its impact on transcriptional regulation, the expression of RUNX1-ETO is not sufficient for the induction of leukemia in transgenic mice, but additional mutations are indispensable for the onset of AML [24,25]. The relevance of RUNX1-ETO for cellular processes, including proliferation, differentiation and cell survival has been investigated in numerous studies. Loss-of-function studies have mostly been carried out by transient, RNAi-mediated silencing of RUNX1-ETO in t(8;21) positive cell lines. Transient depletion of RUNX1-ETO in Kasumi-1 and SKNO-1 has been shown to reduce the proliferation and colony formation, and to restore myeloid differentiation, as measured by expression of *CD11B* and *C/EBPα* [26]. Furthermore, Kasumi-1 cells display a block in transition from the G1- to S-phase of the cell cycle, accompanied by reduced apoptosis and the induction of cellular senescence, in response to RUNX1-ETO RNAi [27]. In line with these findings, Dunne et al. [28] have demonstrated the impact of RUNX1-ETO silencing on the expression of genes associated with proliferation and differentiation, such as Insulin-like growth factor-binding protein 7 (*IGFBP7*) and Cathepsin G (*CTSG*), in Kasumi-1 and patient derived AML blasts with t(8;21) by oligonucleotide array and qRT-PCR.

The aim of our study was to analyze phenotype, target gene expression and potential molecular mechanisms involved in Kasumi-1 cells upon stable long-term silencing of RUNX1-ETO. Unexpectedly, we observed RUNX1-ETO specific differential protein expression of target genes depending on cell lysis conditions. Focusing on this phenomenon, we demonstrate the induction of *CTSG* in response to RUNX1-ETO knockdown to be partially responsible for *in vitro* degradation of cellular proteins. We used this phenomenon to map the activation of proteases beside CTSG in RUNX1-ETO depleted Kasumi-1 cells, as *CTSG* is a well-known target of RUNX1-ETO but not much is known about the possible participation of other proteases. A liquid chromatography (LC)-mass spectrometry (MS)-based approach was applied to measure proteolytic cleavage in RUNX1-ETO silenced Kasumi-1 cells on a proteome-wide scale. The mapping of protease cleavage sites demonstrated a contribution of Neutrophil Elastase (ELANE) to the degradation of cellular proteins in RUNX1-ETO-depleted Kasumi-1 cells, which was functionally confirmed by *ELANE*-specific RNAi.

## Materials and methods

### Cell culture, lentiviral transduction and inhibitors

Kasumi-1 cells (Leibniz-Institute DSMZ, Braunschweig, Germany) are a myeloid cell line carrying the RUNX1-ETO t(8;21) translocation and were cultured in RPMI 1640 supplemented with 10% FCS (Sigma, St. Louis, MO, USA) and 1% Penicillin/Streptomycin (Gibco, Waltham, MA, USA). HEK293 cells (Leibniz-Institute DSMZ) for production of lentiviral particles were maintained in DMEM (Gibco), containing 10% FCS and 1% Penicillin/Streptomycin. Preparation of recombinant lentiviral supernatants and lentiviral transductions were performed as described earlier [29]. Transduction efficacy was determined by flow cytometry (Cytoflex, Beckman Coulter, Brea, CA, USA) based on expression of the lentivirally encoded EGFP or RFP reporters. FlowJo Single Cell Analysis Software v10 was used for data analysis. Cell lines were treated with different inhibitors as indicated. ABT-737 and Bortezomib were purchased from Selleckchem (Houston, TX, USA), Doxorubicin and Chloroquine from Sigma.

### Lentiviral constructs and si/shRNA

The lentiviral transgene plasmids pdcH1-shRNA-SEW/SR and the control plasmids pdcH1-gl4-SEW/SR were cloned as described [29]. The sequence of RUNX1-ETO targeting siRNA was described by Heidenreich et al. in 2003 [26] and converted to an shRNA. ELANE-specific siRNA was obtained from Dharmacon (Lafayette, CO, USA). Cathepsin G targeting shRNA was cloned as described [29]. Oligonucleotides for Cathepsin G shRNA were purchased from BioSpring (Frankfurt am Main, Germany) and the sequences are listed in S1 Table.

### Proliferation and clonogenic assay

Transduced Kasumi-1 cells were cultured at 2x10^5^ cells/ml in 24-well plates, and viable cells were counted by trypan blue exclusion to determine their proliferation rate. For colony assays, Kasumi-1 cells were seeded to 96-well plates in a limiting dilution of one cell per well, five days after lentiviral transduction. Colonies were counted on day 14 after transduction.

### Flow cytometry for apoptosis and cell cycle analysis

Apoptotic cells were quantified by staining with 10 μg/ml Propidium iodide (PI, Serva, Heidelberg, Germany). PI positive cells were considered apoptotic and the percentage of PI positive cells was determined by flow cytometry. Cell cycle distribution of Kasumi-1 cells was analyzed by DNA content. Cells were fixed with 70% ethanol for 24 hours at -20°C and treated with 16.7 μg/ml PI and 20 μg/ml RNaseA in PBS for 30 minutes in the dark. Flow cytometry was performed on a FACSCalibur (Becton Dickinson, Heidelberg, Germany) and BD CellQuest^™^ Pro Software was used for data anlaysis.

### MTS-Assay

The cytotoxicity of ABT-737 on transduced Kasumi-1 cells was determined by CellTiter 96^®^ AQueous One Solution Cell Proliferation Assay (Promega, Madison, WI, USA) according to the manufacturer´s instructions. Briefly, cells were seeded on 96-well plates to a concentration of 2x10^4^/well in culture medium, with ABT-737 added to the indicated concentrations. After 48 hours, cells were incubated with MTS reagent for four hours, followed by measurement of the absorbance at 490 nm on a 96-well plate reader (Mithras LB940, Berthold Technologies, Bad Wildbad, Germany).

### Electron microscopy

Preparation and imaging was done as described in Dawodu et al. [30].

### Western Blot and Coomassie staining

For Western Blot analysis, whole cell lysates were prepared with high-salt lysis buffer (20 mM HEPES, pH 7.5, 0.4 M NaCl; 1 mM EDTA, 1 mM EGTA, 1 mM DTT), as recently described [31]. Alternatively, cells were pelleted and immediately boiled in SDS buffer (2x Laemmli: 62.5 mM Tris pH 6,8, 2% SDS, 10% glycerol, 5% β-mercaptoethanol, 0.001% Bromophenol blue) for five minutes. Prior to boiling, cell lysates were kept on ice at all times. Proteins were separated by SDS-PAGE and transferred to Nitrocellulose (Thermo Fisher Scientific, Waltham, MA, USA) membrane at 800 mA for two hours. Membranes were blocked in 3% non-fat dry milk (Merck, Darmstadt, Germany) for one hour and incubated overnight at 4°C with primary antibodies, followed by one hour incubation with HRP-conjugated secondary antibodies at room temperature. Primary antibodies for RUNX1, GAPDH, LC3A/B and STAT3 were purchased from Cell Signaling Technology (Danvers, MA, USA), antibodies for PU.1 (T-21), STAT5A (L-20) and Ubiquitin from Santa Cruz Biotechnology (Dallas, TX, USA), Cathepsin G antibody from ProteinTech (Rosemont, IL, USA), anti-BCL2 from BD Pharmingen (Franklin Lakes, NJ, USA) and Human Neutrophil Elastase antibody (Clone #950317) from R&D Systems (Minneapolis, MN, USA). Secondary antibodies were purchased from Cell Signaling Technology (anti-rabbit), Santa Cruz (anti-mouse) and BD Pharmingen (anti-hamster). Proteins were visualized on Amersham Hyperfilm^™^ ECL (GE Healthcare) by chemiluminescence using Western Lightning Plus-ECL (Perkin Elmer, Waltham, MA, USA).

For Coomassie staining, proteins were separated by SDS-PAGE, incubated with Coomassie staining solution (0,25% Coomassie Brilliant Blue, 45,5% methanol, 9,2% acetic acid) for 15 minutes at room temperature and destained overnight in destaining solution (30% methanol, 10% acetic acid).

### Quantitative real-time PCR

Total RNA was extracted using Trizol reagent (Invitrogen) and reverse transcription of 1 μg total RNA was performed with M-MLV reverse transcriptase (Invitrogen) and random hexamer primers (Thermo Scientific). SYBR green master mix or Taqman Universal master mix (Applied Biosystems, Foster City, CA, USA) was used for real-time PCR according to the manufacturer´s instructions and reactions were run on an ABI7500 cycler (Applied Biosystems). Expression of mRNA was normalized to 18S rRNA or β2M as housekeeping control and relative expression levels are shown as 2^-ΔΔCT^compared to control cells. Primers and probes were synthetized by BioSpring, except for STAT5 primers and probe, which were purchased by MWG Biotech (Ebersberg, Germany). Taqman Assays were purchased from Applied Biosystems. Sequences of primers and probes are listed in S1 Table.

### Sample preparation for MS analysis

Whole cell lysates from Kasumi-1/ctrl and Kasumi-1/shRE were prepared in RIPA buffer (50mM Tris HCl pH 7.5, 150 mM NaCl, 5 mM EDTA, 1% Triton X-100, 0.5% sodium deoxycholate, 0.1% SDS), supplemented with complete Protease Inhibitor Cocktail (Roche), on day twelve after lentiviral transduction. Protein concentration was determined by DC-Assay (Bio-Rad Laboratories, Hercules, CA, USA) according to the manufacturer’s instructions and 50 μg protein per sample were boiled for five minutes in 2x NuPage LDS sample buffer (Invitrogen). Proteins were alkylated by acrylamide and further processed as described [32]. Peptide samples were analyzed with data dependent analysis in an LC-MS system (RSLC, LTQ Orbitrap Velos, both Thermo Fisher) as described recently [32]. Raw MS data were processed using Proteom discoverer 1.4 (Thermo Scientific) and Max Quant software (version 1.5, Cox and Mann 2008) and reviewed human and viral entries of the SwissProtUniprot database containing common contaminants. Proteins were stated identified by a false discovery rate of 0.01 on protein and peptide level.

### Statistics

GraphPad Prism (version 7.04) has been used for statistical analyses by student´s t-test or 2-way ANOVA, as indicated. Statistical significance was considered for p<0.05.

## Results and discussion

### Stable long-term RUNX1-ETO silencing in Kasumi-1 cells

**Fig 1:**
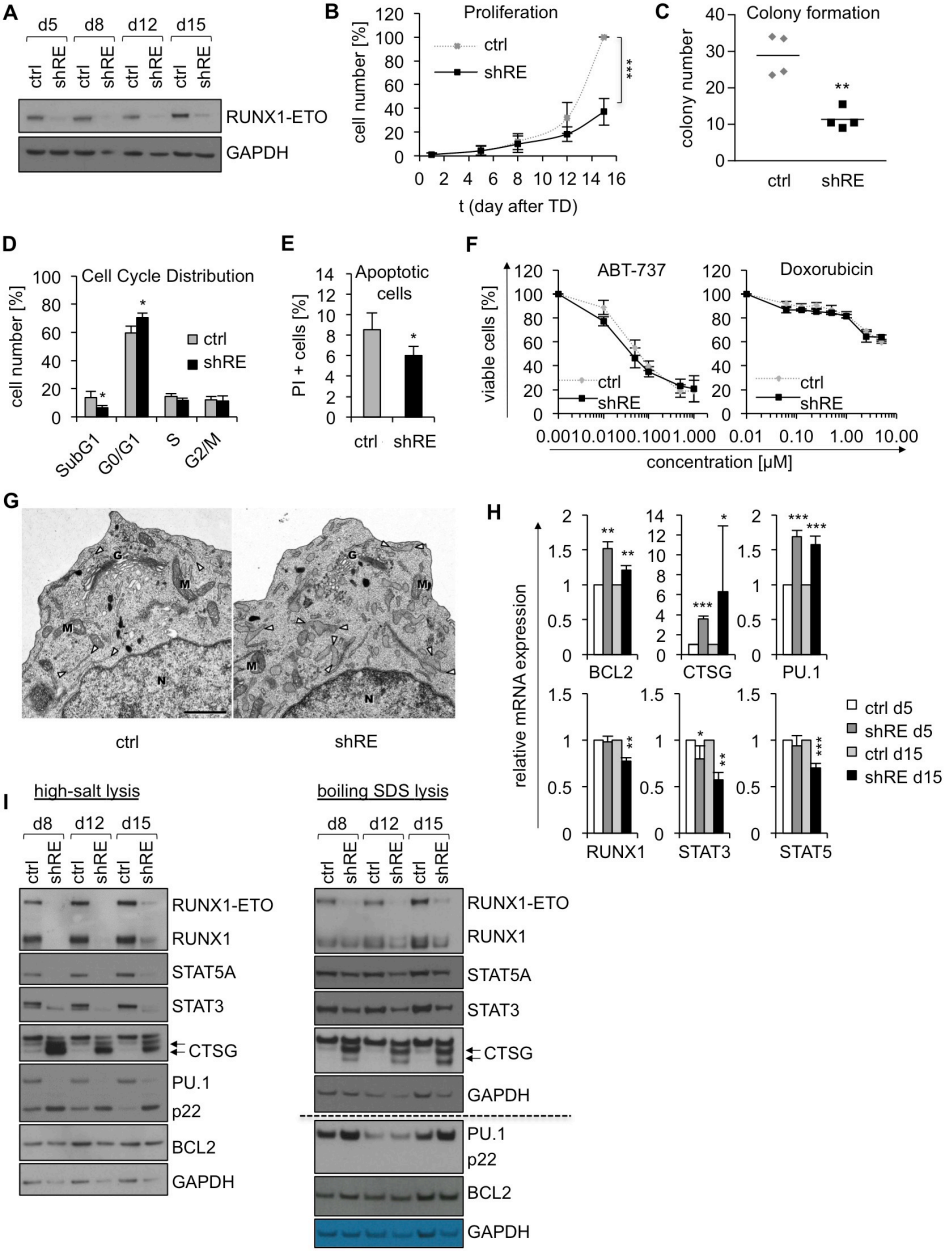
Phenotype of RUNX1-ETO knockdown and kinetics of protein/mRNA expression in Kasumi-1. A) Kasumi-1 cell lysates were prepared in SDS buffer at the indicated time points post transduction and RUNX1-ETO depletion was confirmed by Western Blot (n = 3). B) Cell expansion of Kasumi-1/ctrl and Kasumi-1/shRE was determined by counting and trypan blue exclusion, and growth curves depict cell numbers relative to control cells at day 15 after lentiviral transduction (n = 4). C) Number of Kasumi-1/ctrl and Kasumi-1/shRE colonies, as counted nine days after plating in limiting dilution (n = 4). Cell cycle distribution (D) of Kasumi-1/ctrl and Kasumi-1/shRE and the percentage of apoptotic cells (E) were determined by PI staining and subsequent flow cytometry at day twelve after transduction (n = 4). F) The response of Kasumi-1/ctrl and Kasumi-1/shRE to ABT-737 and Doxorubicin was measured by MTS Assay and PI staining, respectively, at day 14 post transduction (n = 3). The percentage of viable cells is shown relative to untreated cells after 48 hours of treatment with the indicated drug concentrations. G) Transmission electron microscopy of Kasumi-1/ctrl (left) and Kasumi-1/shRE (right) cells, representative for three independent experiments. N, nucleus; G, Golgi; M, mitochondria. Profiles of ER cisternae are highlighted by arrowheads. Note the flat and evenly formed ER cisternae in Kasumi-1/ctrl, compared to frequently dilated cisternae in Kasumi-1/shRE cells. Scale bar = 1 μm. Protein and mRNA expression of RUNX1-ETO target genes were analyzed at the indicated time points after lentiviral transfer of RUNX1-ETO-specific shRNA. H) mRNA expression was determined relative to control cells and normalized to housekeeping control. I) Whole-cell lysates of Kasumi-1/ctrl and Kasumi-1/shRE were prepared in high-salt lysis buffer (left, n = 4) or boiling SDS buffer (right, n = 3) and analyzed by Western Blot. Dashed line indicates separate membranes. Data are represented as mean +/- SD and p-values were calculated using 2-way ANOVA (B+F) or two-sided student´s t-test (C,D,E,H). *p<0.05, **p<0.01, ***p<0.001.

Stable knockdown of RUNX1-ETO was achieved by lentiviral transduction of Kasumi-1 cells with RUNX1-ETO specific shRNA. RUNX1-ETO-silenced (Kasumi-1/shRE) and control cells (Kasumi-1/ctrl) were monitored over 15 days for phenotypic changes and alterations in protein and mRNA expression. Transduction efficacy was determined based on eGFP reporter fluorescence and consistently reached >96% (Table A in S1 Fig). Stable depletion of RUNX1-ETO was verified by Western Blot (Fig 1A). Cell expansion of Kasumi-1/shRE cells was reduced as compared to Kasumi-1/ctrl cells (Fig 1B), and clonal expansion of Kasumi-1/shRE cells plated in limiting dilution was also decreased (Fig 1C), as described previously [26]. In line with the study of Martinez et al. 2004 [27], cell cycle analysis revealed a partial G0/G1 arrest and a smaller SubG1 fraction in Kasumi-1/shRE compared to Kasumi-1/ctrl cells (Fig 1D). These findings were supported by a reduced percentage of apoptotic Kasumi-1/shRE cells in comparison to control cells, as observed by PI staining (Fig 1E). However, RUNX1-ETO-depleted Kasumi-1 and control cells displayed equal sensitivity to the anthracycline doxorubicin and the BH3 mimetic ABT-737, which triggers mitochondrial apoptosis (Fig 1F).

Electron microscopy revealed dilated cisternae in most of all cell profiles in Kasumi-1/shRE, in contrast to control cells (n = 3) (Fig 1G). Cisternae were identified as endoplasmatic reticulum (ER) by their arrangement, clearly discernable connections to not dilated parts of ER cisternae and by ribosomes on their surface. No other discernable alterations in cell morphology were detected, including Golgi apparatus, mitochondria, nuclei, cell size or other cellular components or ultrastructural parameters. ER dilation is recognized as a sign of ER stress [33–35] and has not yet been described in the context of RUNX1-ETO loss-of-function. However, the induction of granulocytic differentiation has been linked to ER stress, as a consequence of increased folding demands due to enhanced expression of secretory proteins, in acute promyelocytic leukemia (APL) cell lines and primary blasts [36]. The release of RUNX1-ETO suppression in Kasumi-1 has been shown to induce myeloid differentiation [26] and may present a conceivable explanation for the ER dilation, observed in our study.

Next, we analyzed the kinetics of mRNA and protein expression of RUNX1-ETO target genes up to 15 days after lentiviral transduction of Kasumi-1 cells. As expected from the mode of RUNX1-ETO action, the well-known RUNX1-ETO targets PU.1, BCL2 and CTSG [16,13,28] were upregulated on both mRNA and protein level on day five after transduction (Fig 1H and S1 Fig). Surprisingly, Western Blot analysis of cellular extracts, prepared in high-salt lysis buffer, showed a strong reduction of RUNX1-ETO target protein expression at later time points post transduction (Fig 1I, left), while the mRNA levels remained stable (Fig 1H, upper panel). In contrast, non RUNX1-ETO target genes, such as RUNX1, STAT5A and STAT3 showed a consistent reduction of both mRNA (Fig 1H, lower panel) and protein levels (Fig 1I) throughout the time course. However, such a reduction in protein expression upon stable anti RUNX1-ETO RNAi has not yet been described. In addition to reduced protein expression, we observed a lower molecular weight isoform of PU.1 (referred to as PU.1 p22), immunoreactive with a C-terminal PU.1 antibody, as detected by Western Blot (Fig 1I, left). Expression of PU.1 p22 strongly increased after RUNX1-ETO knockdown in parallel to reduced expression of wildtype PU.1, suggesting proteolytic cleavage of PU.1. However, treatment of the cells with inhibitors of proteasomal (Bortezomib) and lysosomal (Chloroquine) degradation did not rescue protein expression of RUNX1-ETO target genes or reduce the occurrence of PU.1 p22 (S2 Fig). To evaluate the significance of these observations, we analyzed possible cell lysis-dependent effects, applying alternative protocols for the lysis of Kasumi-1/shRE and control cells (S2 Fig). Indeed, preparation of cellular lysates in boiling SDS sample buffer did not result in the observed loss of protein expression and PU.1 p22 was not detected. The resulting protein expression kinetics matched the kinetics of mRNA expression (Fig 1I, right), indicating protein degradation in Kasumi-1/shRE during the preparation of whole cell lysates under high-salt conditions.

The lysosomal protease CTSG is a well-known RUNX1-ETO target [28] and was strongly induced in our experiments (Fig 1H+1I), representing an obvious candidate for the degradation of proteins under cell lysis conditions in our study. CTSG has been shown to degrade RUNX1-ETO *in vitro* and *in vivo* [37]. This process has been discussed as a cellular mechanism to selectively remove aberrant proteins, and the suppression of CTSG by RUNX1-ETO could promote leukemogenesis by evading this intracellular surveillance system [37]. Furthermore, Schuster et al. identified CTSG as the STAT5 protease [38], which has been ascribed to the generation of C-terminally truncated isoforms of STAT5A and STAT5B, referred to as STAT5γ [39–41]. Truncated isoforms have also been observed for STAT3 [42–44] and STAT6 [45–47]. The relevance of STAT5γ has been discussed in the context of apoptosis, myeloid differentiation and AML [48–52]. However, Schuster et al. [38] clearly demonstrated that the generation of STAT5γ does not happen *in vivo*, but is an artifact of cell lysis. This finding might also question the significance of CTSG-mediated cleavage of RUNX1-ETO, which has been discussed by Jin et al. [37]. Based on the above-mentioned studies, the strong induction of CTSG and the loss of detectable STAT3 and STAT5 protein in Kasumi-1/shRE cells (Fig 1H+1I) suggest a contribution of CTSG to the decrease of protein expression in our study.

### Mapping of protease cleavage patterns in RUNX1-ETO silenced Kasumi-1 cells by LC-MS-based peptide analysis

**Fig 2:**
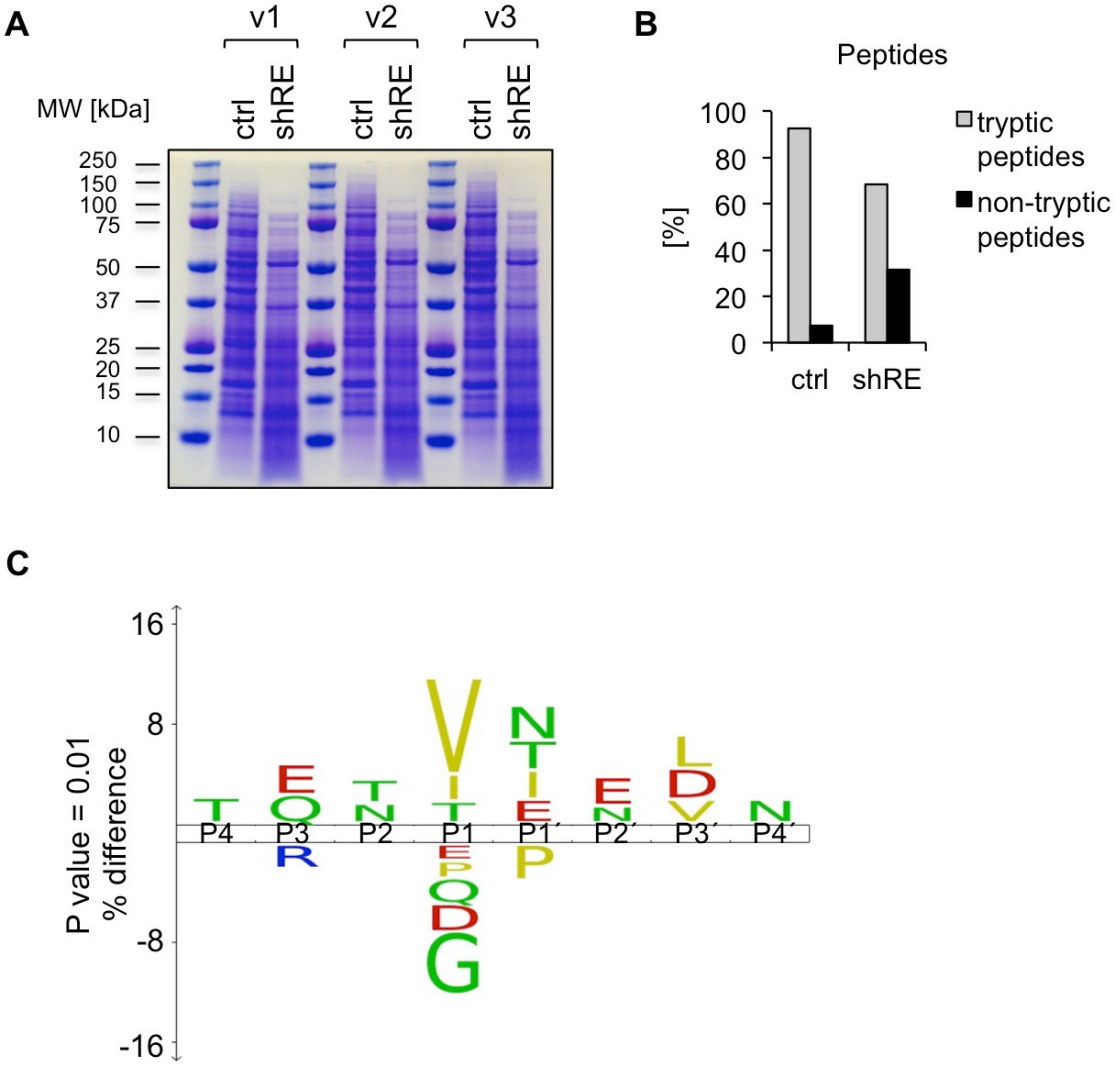
Mapping of protease cleavage sites by mass spectrometry. A) Whole cell lysates of Kasumi-1/ctrl and Kasumi-1/shRE cells were prepared in RIPA buffer supplemented with cOmplete Protease Inhibitor Cocktail (Roche) on day twelve after transduction. Proteins were separated by SDS PAGE, stained with Coomassie blue (n = 3) and subjected to LC-MS analysis. B) The identified peptides were categorized as tryptic peptides, resulting from trypsin digestion during the MS sample preparation procedure, or non-tryptic peptides, resulting from proteolytic cleavage during cell lysis. C) IceLogo showing amino acids at positions P4-P4´, which are enriched or depleted in peptides from Kasumi-1/shRE compared to Kasumi-1/ctrl with p<0.01. Red, acidic; blue, basic; yellow, nonpolar hydrophobic; green, polar neutral amino acids. Results shown in B) and C) are representative for one of three independent measurements.

To analyze the incidence of proteolytic cleavage and to identify the contributing proteases on a proteome-wide scale, we employed LC-MS proteomics, referring to the approach, described by Gupta et al. 2010 [53] which allows the mapping of proteolytic cleavage sites in any MS dataset. Kasumi-1/shRE and Kasumi-1/ctrl cells were lysed in RIPA buffer, supplemented with commercially available Protease Inhibitor Cocktail, and separated by SDS-PAGE. The pattern of the protein bands after Coomassie staining indicates increased proteolytic activity in the Kasumi-1/shRE lysates (Fig 2A) in line with our previous observation of RUNX1-ETO knockdown resulting in protein degradation in Kasumi-1 cells (Fig 1I). The proteins were excised from the gel, trypsinized and subjected to LC-MS analysis. Trypsin is a highly specific protease, cleaving only after Lysine (K) or Arginine (R). Thus, we were able to differentiate between peptides, which can be ascribed to trypsin digestion as a part of the procedure for MS sample preparation (tryptic peptides), and peptides resulting from proteolysis by other proteases, either due to endogenous proteolytic activity, or during cell lysis (non-tryptic peptides). Peptides with K or R at position P1 of their N- and C-terminal ends were considered tryptic, while all peptides with at least one end being not K or R and the other end being not the first or the last amino acid of the protein, were considered to be products of proteolytic cleavage during cell lysis. Based on this analysis, we identified a total of 17,490 peptides, with 5767 and 3370 peptides being unique to Kasumi-1/ctrl and Kasumi-1/shRE, respectively. We found 7.4% of the peptides from Kasumi-1/ctrl but 31.5% of the peptides from Kasumi-1/shRE to be of non-tryptic origin, which reflects a more than 4-fold increase of proteolytic activity in Kasumi-1/shRE compared to control cells (Fig 2B).

In a next step, we looked at the distribution of the amino acids surrounding the cleavage sites of the non-tryptic peptides to assign the responsible proteases. The amino acid P4-P4´ of peptides derived from Kasumi-1/ctrl and Kasumi-1/shRE have been integrated into an IceLogo [54] to identify conserved sequence patterns (Fig 2C). The IceLogo shows a prominent enrichment of the hydrophobic amino acids Valine (V), Isoleucine (I) and Threonine (T) in position P1, representing the amino acid before the cleavage site. In addition, a noticeable over-representation of the acidic amino acids Glutamate (E) and Aspartate (D) in the positions P1´-P3´, downstream of the cleavage site has been observed. To draw conclusions from the proteolytic signature in our dataset to the corresponding proteases, we searched the literature and the Peptidase Database Merops (http://www.ebi.ac.uk/merops/) [55] for established protease cleavage patterns. As the lysosomal protease CTSG is a well-known target of RUNX1-ETO and strongly induced by stable anti-RUNX1-ETO RNAi in our experiments (Fig 1H+1I) we focused on lysosomal enzymes. According to O´Donoghue et al. [56], CTSG has a preference for the aromatic amino acids Phenylalanine (F) and Tyrosine (Y) in position P1 and does not match the observed cleavage patterns in our dataset. However, we did find overlaps between the proteolytic signatures of Kasumi-1/shRE peptides and the signature for Neutrophil Elastase (ELANE), which shows enrichment for Isoleucine (I), Valine (V) and Threonine (T) at position P1 of its substrates, as shown by substrate specificity profiling [56]. In addition, Vizovisek et al. [57] have demonstrated an over-representation of Glutamate (E) and Aspartate (D) at positions P1´-P4´ of cleavage sites detected by Cathepsins K, L and S (CTSK/L/S), which also fits the cleavage site pattern observed in our study.

### ELANE and CTSG contribute to cell lysis-induced protein degradation in response to long-term RUNX1-ETO silencing in Kasumi-1 cells

**Fig 3:**
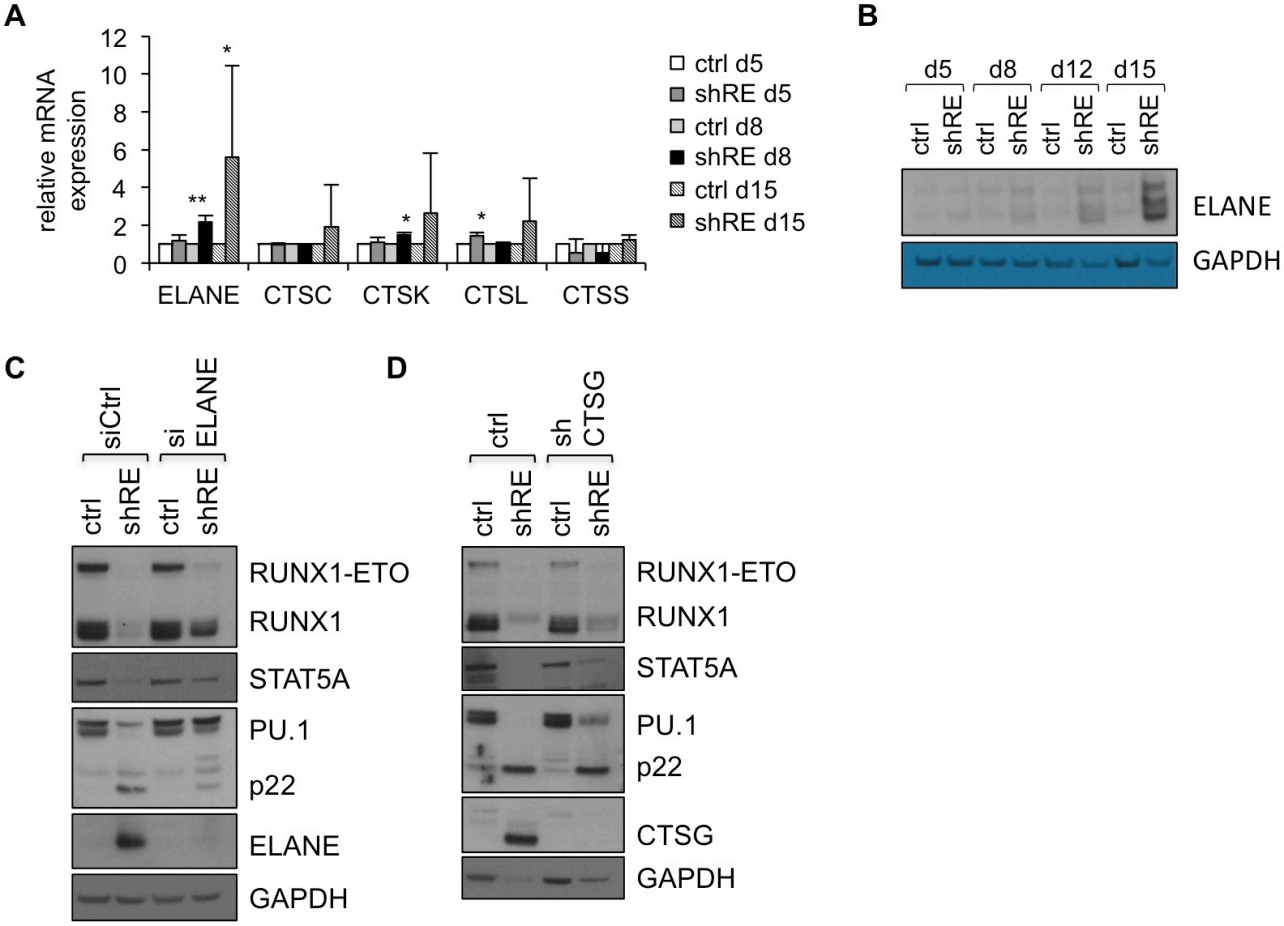
ELANE and CTSG contribute to cell lysis-induced protein degradation in RUNX1-ETO-depleted Kasumi-1 cells. A) RUNX1-ETO dependent mRNA expression of ELANE and CTSC/K/L/S in Kasumi-1 cells was determined by qRT-PCR and normalized to housekeeping control. Data are shown as mean 2^-ΔΔCT^ +/- SD (n = 3) and p-values were calculated by two-sided student´s t-test (*p<0.05, **p<0.01). B) Whole cell lysates of Kasumi-1/shRE and control cells have been prepared in SDS sample buffer and analyzed for ELANE protein expression by Western Blot. C) Kasumi-1/ctrl and Kasumi-1/shRE cells were consecutively electroporated with ELANE-specific or control siRNA on days five and eight after lentiviral transduction with control virus or shRE. Whole cell lysates were prepared in high-salt lysis buffer on day eleven after RUNX1-ETO depletion and protein expression was analyzed by Western Blot. D) Kasumi-1 cells were transduced with CTSG-specific shRNA (Kasumi-1/shCTSG) or control virus (Kasumi-1/ctrl), followed by transduction with RUNX1-ETO-specific shRNA or control virus, two days after the first lentiviral transduction. Whole cell lysates were prepared in high-salt lysis buffer on day twelve after RUNX1-ETO knockdown (respectively day 14 after CTSG knockdown) and analyzed by Western Blot. Western Blots are representative for three independent experiments.

To narrow down the spectrum of proteases involved in protein degradation in RUNX1-ETO-silenced Kasumi-1 cells, we examined RUNX1-ETO-dependent expression of *ELANE* and *CTSK/L/S* in Kasumi-1 by qRT-PCR (Fig 3A). Cathepsin C (CTSC, Dipeptidyl-peptidase I) has been included in our analysis, as it is a common activator of CTSG and ELANE [58]. Only a weak induction was observed for *CTSC* and *CTSK/L/S* in response to RUNX1-ETO silencing in Kasumi-1 cells, while the expression of *ELANE* mRNA was clearly enhanced at the later time points after RUNX1-ETO knockdown. In addition, upregulation of ELANE protein expression in Kasumi-1/shRE has been confirmed by Western Blot (Fig 3B). A possible direct regulation of *ELANE* by RUNX1-ETO has already been excluded by Lausen et al. 2006 [59], but *ELANE* expression is controlled by hematopoietic transcription factors, such as the well-known RUNX1-ETO targets C/EBPα and PU.1 [60,61]. This suggests an indirect induction of *ELANE* expression in response to the release of C/EBPα and PU.1 suppression in Kasumi-1/shRE.

The combined results of the gene expression analysis and the consultation of available protease cleavage patterns suggest a contribution of ELANE to the proteolytic activity observed in Kasumi-1/shRE. As a consequence, depletion of ELANE in Kasumi-1 should rescue protein expression after RUNX1-ETO knockdown. Indeed, repeated electroporation of Kasumi-1/ctrl and Kasumi-1/shRE with ELANE-specific siRNA partially reversed cell lysis-triggered degradation of RUNX1, STAT5A and PU.1, and diminished detection of PU.1 p22 in Kasumi-1/shRE (Fig 3C). Although the proteolytic cleavage patterns described for CTSG did not resemble the proteolytic signature generated from our dataset, we still considered a contribution of CTSG to the degradation of proteins in RUNX1-ETO-silenced Kasumi-1 cells. Expression of *CTSG* was strongly induced in response to RUNX1-ETO depletion in our experiments (Fig 1H+1I). Furthermore, CTSG has been described to cleave STAT5 in a cell lysis-dependent mode of action [38], and we found the protein levels of STAT5A to be decreased in Kasumi-1/shRE (Fig 1I), dependent on the cell lysis procedure (S2 Fig). Consequently, we investigated a possible contribution of CTSG to the cell lysis-induced protein degradation in Kasumi-1/shRE by shRNA-mediated depletion of CTSG in Kasumi-1 cells, followed by knockdown of RUNX1-ETO. Indeed, silencing of *CTSG* expression diminished protein degradation in Kasumi-1/shRE during the preparation of whole cell lysates, as shown for RUNX1, STAT5A, PU.1 and GAPDH (Fig 3D). In contrast to ELANE, CTSG does not seem to contribute to the generation of PU.1 p22. Knockdown of *ELANE* and *CTSG* was confirmed by Western Blot (Fig 3C+3D) and qRT-PCR (S3 Fig). Finally, the most right lane in Fig 3C and 3D demonstrates specific silencing of ELANE and CTSG by RNAi, respectively.

Taken together, we describe a mechanism by which the high abundance of lysosomal proteases in combination with high-salt cell lysis conditions leads to the degradation of cellular proteins during the preparation of whole cell lysates. This phenomenon has led to false interpretation of protein expression data before as in case of the intensely discussed STAT5γ isoform which has been shown to be generated by CTSG during the preparation of nuclear extracts [38]. This might present a major pit fall for the investigation of protein expression under conditions which promote proteolytic activity such as the increased expression of lysosomal proteases in response to RUNX1-ETO depletion.

Silencing of RUNX1-ETO in t(8;21) positive cells has been shown to promote granulocytic differentiation accompanied by the expression of lysosomal proteins [26,28]. As a consequence, the upregulation of lysosomal proteases may also compromise proteomic studies concerning leukemic differentiation.

Using LC-MS data and sequence analysis of peptide cleavage sites, we propose the lysosomal proteases ELANE and CTSK/L/S as possible candidates for the degradation of cellular proteins in Kasumi-1/shRE triggered by cell lysis. The contribution of ELANE and CTSG was confirmed by si/shRNA knockdown although the LC-MS analysis did not show any footprints of CTSG activity on Kasumi-1/shRE derived peptides. This may be due to limited sensitivity of this screening approach. The contribution of CTSK/L/S has not been further evaluated, as we observed only a weak induction in response to RUNX1-ETO silencing in Kasumi-1 cells. However, the regulation of lysosomal proteases is not limited to gene expression, but relies on a complex network of activation cascades including the proteolytic processing of inactive zymogens [62–64] and the regulation of endogenous protease inhibitors. For instance, CTSG and ELANE are able to activate Cathepsin B [65], which in turn activates CTSL via CTSD [66]. The endogenous inhibitor of CTSK and CTSL, Serpin B13 [67], is induced by RUNX1 [68] and, hypothetically, could be a possible target for RUNX1-ETO suppression, leading to an increase in CTSK and CTSL activity in Kasumi-1/shRE cells.

Our data demonstrate that the protein degradation in response to long-term RUNX1-ETO RNAi does not occur *in vivo*, but depends on cell lysis conditions which allow the release of proteases from the lysosomes. Yet, it cannot be excluded, that the strong induction of proteolytic activity by RUNX1-ETO depletion has any impact on the cells and their environment *in vivo*, as the functions of CTSG and ELANE are not restricted to the lysosomal compartment, but can be secreted [69–71] to modulate the activity of cytokines and their receptors [72].

## Supporting information

S1 Table Taqman assays and sequences of primers and probes for qRT-PCR. Oligonucleotides used for cloning of CTSG-specific shRNA with underlined target sequence.
(TIF)

S1 Fig Target gene expression in RUNX1-ETO silenced Kasumi-1 cells. A) Transduction efficacy of lentivirally transduced Kasumi-1 cells was determined by flow cytometry based on GFP-reporter fluorescence. B) Whole cell lysates were prepared in high-salt lysis buffer on day 5 after lentiviral transduction and expression of RUNX1-ETO target genes was determined by Western Blot (n = 4).
(TIF)

S2 Fig Decreased protein levels in Kasumi-1/shRE cannot be restored by inhibition of proteasomal and lysosomal degradation. Kasumi-1/ctrl and Kasumi-1/shRE cells were treated with Bortezomib (A) or Chloroquine (B) on day 13 after lentiviral transduction, as indicated. Whole cell lysates were analyzed for the expression of RUNX1-ETO target genes by Western Blot. Proteasomal and lysosomal inhibition was confirmed by detecting accumulation of ubiquitinylated proteins or processing of the autophagy marker LC3A/B (microtubule-associated proteins 1A/1B light chain 3B), respectively. PI staining was performed to evaluate cytotoxicity of the different inhibitors by flow cytometry and the percentage of PI-positive cells is shown at the bottom of each Western Blot. Data are representative for three independent experiments. C) Lysates of Kasumi-1/ctrl and Kasumi-1/shRE cells were prepared at day 14 after shRNA-mediated RUNX1-ETO knockdown and analyzed by Western Blot. The application of different lysis conditions demonstrates the impact of the cell lysis procedure on protein stability in RUNX1-ETO-silenced Kasumi-1 cells. Data are representative for one of three independent experiments.
(TIF)

S3 Fig Confirmation of si/shRNA-mediated knockdown of ELANE and CTSG by qRT-PCR. ELANE and CTSG mRNA levels were measured by qRT-PCR and normalized to housekeeping control. Data are shown as log2 of mean 2^-ΔΔCT^ +/- SD and p-values were determined by two-sided student´s t-test. *p<0.05, ***p<0.001.
(TIF)
