# Supplementary figures and images for: Stable depletion of RUNX1-ETO in Kasumi-1 cells induces expression and enhanced proteolytic activity of Cathepsin G and Neutrophil Elastase
Source: PLoS One. 2019 Dec 11;14(12):e0225977. doi: 10.1371/journal.pone.0225977 (PMC6905530; doi:10.1371/journal.pone.0225977)

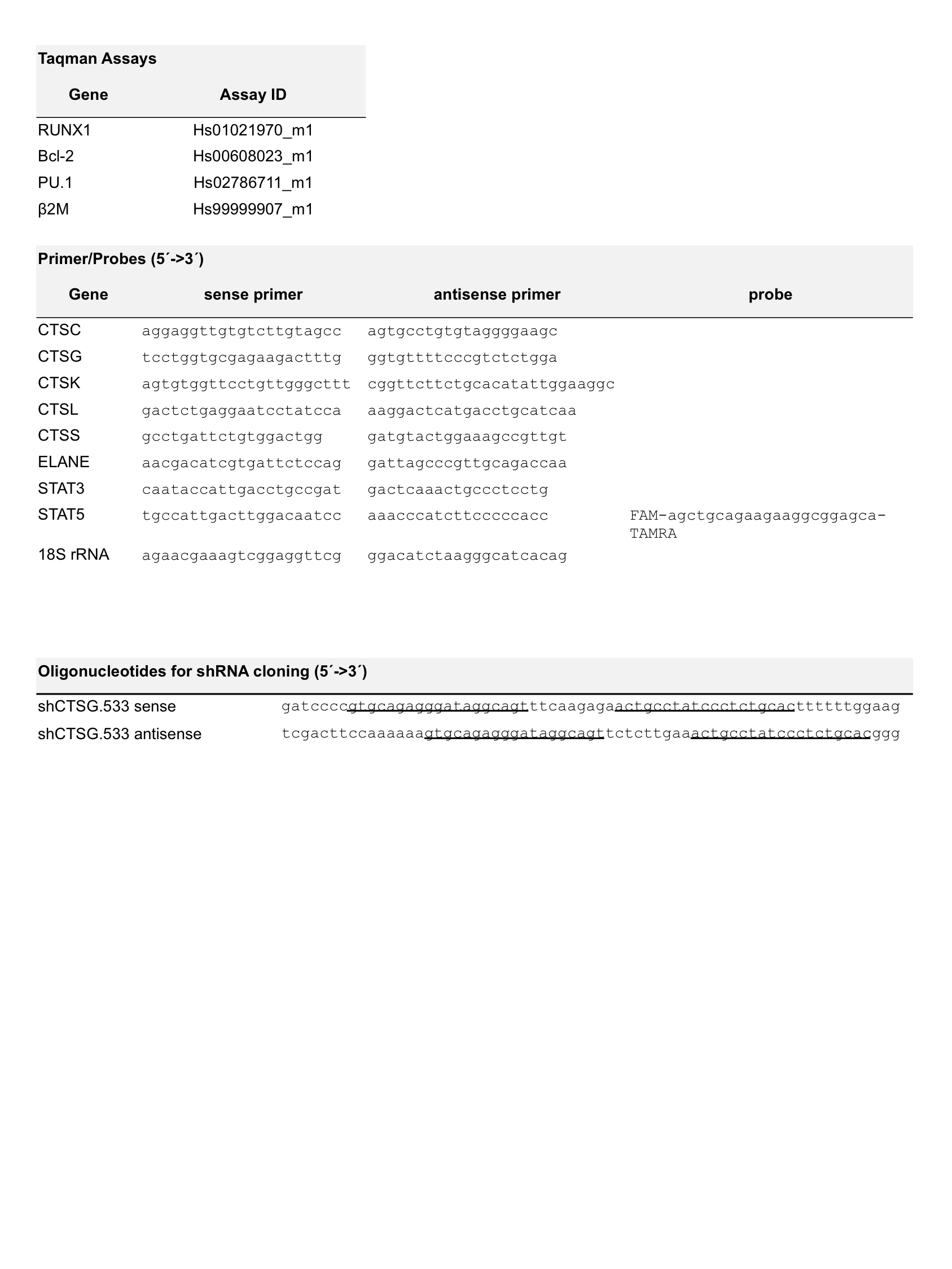

Supplement: S1 Table — Oligonucleotides used for cloning of CTSG-specific shRNA with underlined target sequence. (TIF) [file pone.0225977.s001.tif]

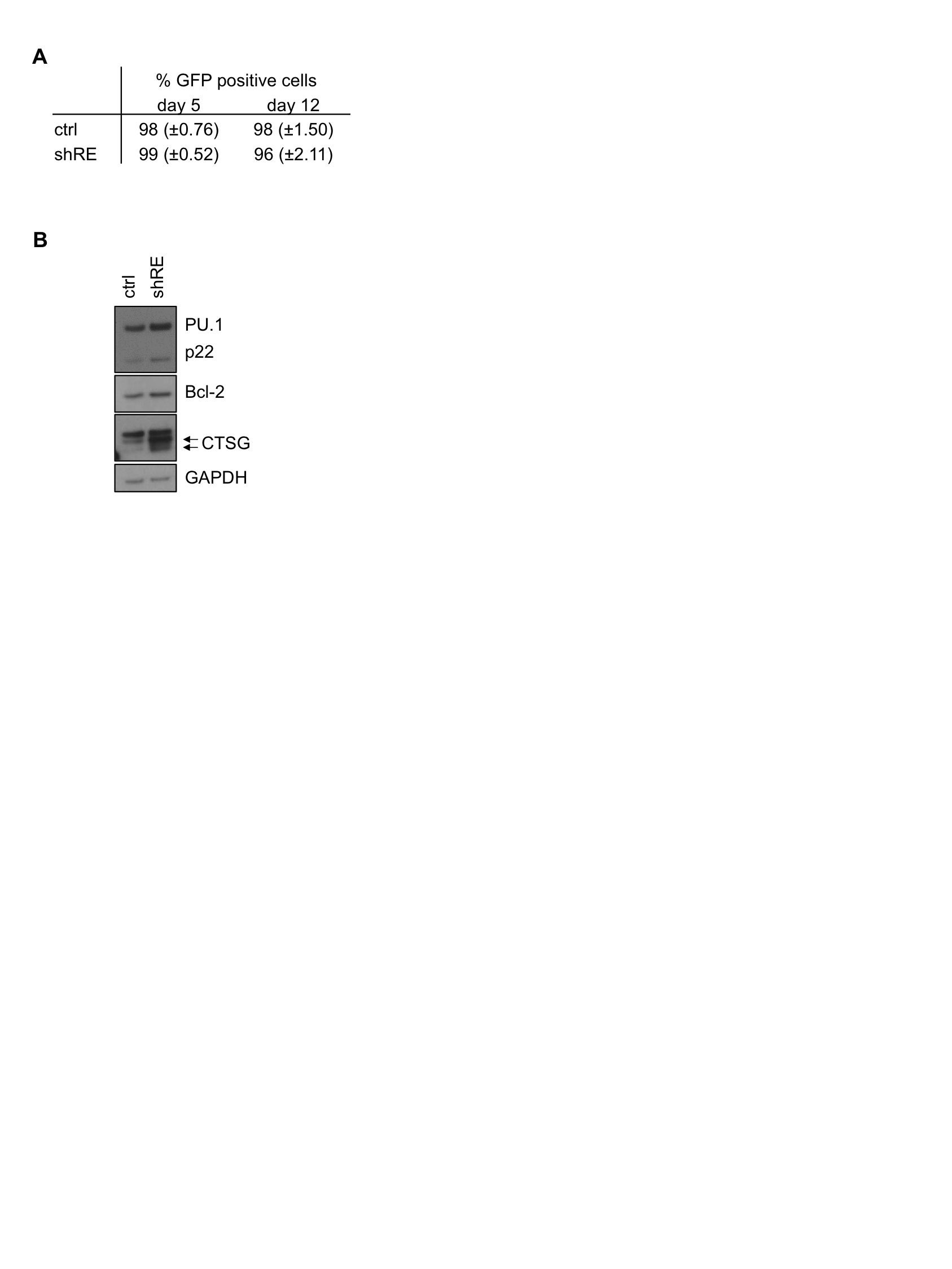

Supplement: S1 Fig — A) Transduction efficacy of lentivirally transduced Kasumi-1 cells was determined by flow cytometry based on GFP-reporter fluorescence. B) Whole cell lysates were prepared in high-salt lysis buffer on day 5 after lentiviral transduction and expression of RUNX1-ETO target genes was determined by Western Blot (n = 4). (TIF) [file pone.0225977.s002.tif]

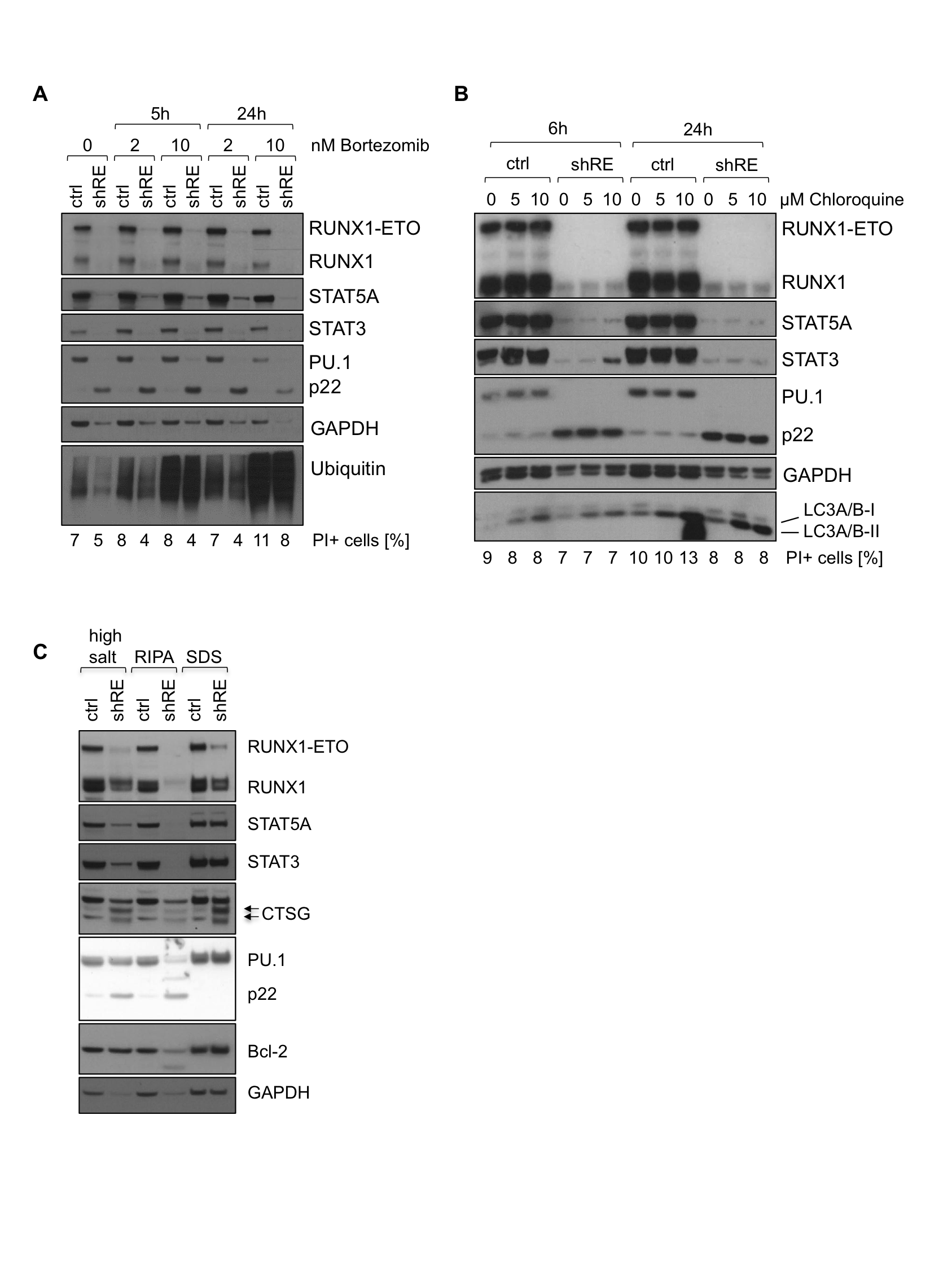

Supplement: S2 Fig — Kasumi-1/ctrl and Kasumi-1/shRE cells were treated with Bortezomib (A) or Chloroquine (B) on day 13 after lentiviral transduction, as indicated. Whole cell lysates were analyzed for the expression of RUNX1-ETO target genes by Western Blot. Proteasomal and lysosomal inhibition was confirmed by detecting accumulation of ubiquitinylated proteins or processing of the autophagy marker LC3A/B (microtubule-associated proteins 1A/1B light chain 3B), respectively. PI staining was performed to evaluate cytotoxicity of the different inhibitors by flow cytometry and the percentage of PI-positive cells is shown at the bottom of each Western Blot. Data are representative for three independent experiments. C) Lysates of Kasumi-1/ctrl and Kasumi-1/shRE cells were prepared at day 14 after shRNA-mediated RUNX1-ETO knockdown and analyzed by Western Blot. The application of different lysis conditions demonstrates the impact of the cell lysis procedure on protein stability in RUNX1-ETO-silenced Kasumi-1 cells. Data are representative for one of three independent experiments. (TIF) [file pone.0225977.s003.tif]

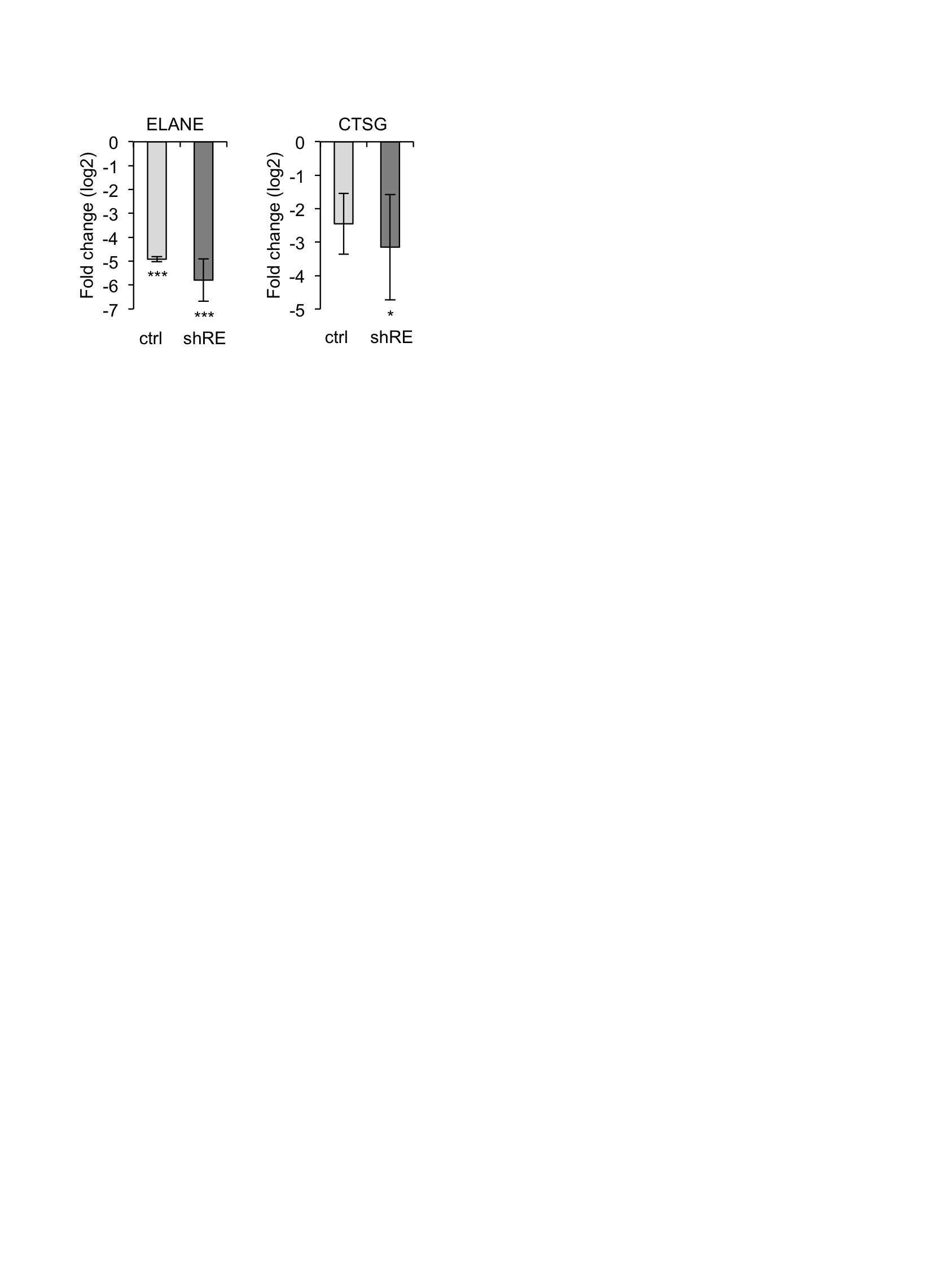

Supplement: S3 Fig — ELANE and CTSG mRNA levels were measured by qRT-PCR and normalized to housekeeping control. Data are shown as log2 of mean 2-ΔΔCT +/- SD and p-values were determined by two-sided student´s t-test. *p<0.05, ***p<0.001. (TIF) [file pone.0225977.s004.tif]
